# Gait Rhythm Dynamics for Neuro-Degenerative Disease Classification via Persistence Landscape- Based Topological Representation

**DOI:** 10.3390/s20072006

**Published:** 2020-04-03

**Authors:** Yan Yan, Kamen Ivanov, Olatunji Mumini Omisore, Tobore Igbe, Qiuhua Liu, Zedong Nie, Lei Wang

**Affiliations:** 1 Shenzhen Institutes of Advanced Technology, Chinese Academy of Sciences, No. 1068 Xueyuan Avenue, Shenzhen University Town, Shenzhen 518055, China; yan.yan@siat.ac.cn (Y.Y.); kamen@siat.ac.cn (K.I.); omisore@siat.ac.cn (O.M.O.); victor@siat.ac.cn (T.I.); qh.liu@siat.ac.cn (Q.L.); zd.nie@siat.ac.cn (Z.N.); 2 University of Chinese Academy of Sciences, Beijing 100049, China

**Keywords:** gait analysis, neuro-degenerative analysis, Parkinson’s diseases, topological data analysis, persistence landscape, persistent homology, nonlinear dynamics analysis

## Abstract

Neuro-degenerative disease is a common progressive nervous system disorder that leads to serious clinical consequences. Gait rhythm dynamics analysis is essential for evaluating clinical states and improving quality of life for neuro-degenerative patients. The magnitude of stride-to-stride fluctuations and corresponding changes over time—gait dynamics—reflects the physiology of gait, in quantifying the pathologic alterations in the locomotor control system of health subjects and patients with neuro-degenerative diseases. Motivated by algebra topology theory, a topological data analysis-inspired nonlinear framework was adopted in the study of the gait dynamics. Meanwhile, the topological representation–persistence landscapes were used as input of classifiers in order to distinguish different neuro-degenerative disease type from healthy. In this work, stride-to-stride time series from healthy control (HC) subjects are compared with the gait dynamics from patients with amyotrophic lateral sclerosis (ALS), Huntington’s disease (HD), and Parkinson’s disease (PD). The obtained results show that the proposed methodology discriminates healthy subjects from subjects with other neuro-degenerative diseases with relatively high accuracy. In summary, our study is the first attempt to provide a topological representation-based method into the disease classification with gait rhythms measured from the stride intervals to visualize gait dynamics and classify neuro-degenerative diseases. The proposed method could be potentially used in earlier interventions and state monitoring.

## 1. Introduction

Neuro-degenerative disease is a common progressive disorder of the nervous system, which might lead to the tremor of limbs, jaw or face, and stiffness of slowing of movement [1]. The neuro-degenerative disease symptoms usually emerge slowly and finally cause movement problems and difficulty with walking. While the gait abnormality as a deviation of walking may reflect different disorder patterns, gait analysis is an essential tool to assess neuro-degenerative disease [2,3,4]. In [5], Kamruzzaman uses two basic temporal-spatial gait parameters (stride length and cadence) as input features and support vector machine method to analyze the cerebral palsy gait. The authors of [6] reported multiple regression normalization strategies that incorporated physical properties and self-selected speed for Parkinson’s Disease Gait analysis. In [7], Wu used a nonparametric Paren-window method to estimate the probability density functions of stride interval and its sub-phases: swing interval and stance interval, with the statistical analysis of gait rhythm. In [8], an investigation of using frequency range distribution to gain new insight on gait rhythm and implements fluctuation analysis. In [9], the author proposed a model of tensor decomposition for higher-dimensional analysis in Parkinson’s disease. In [10], the gait 1-D signals are converted into texture-based images to get insights into disease patterns with a fuzzy recurrence plot. Studying the dynamics of gait patterns in neuro-degenerative disease to diagnose the severity, could be conducive to fall prediction, treatment, and rehabilitation strategies improvement. In this paper, we explore the stride-to-stride intervals as the gait-phase representation to study the gait dynamics for neuro-degenerative diseases. The stride-to-stride intervals extracted from the plantar pressure signals form interval-based time series sequences [11], i.e., gait rhythm.

Exploring features from the gait rhythm dynamics and used for disease state recognition is an essential topic in physiological signal analysis. Recently, a powerful tool based on algebra topology theory was proposed for data analysis, which is termed topological data analysis (TDA). TDA techniques have become a useful representation extraction tool for complex data analysis and visualization [12,13,14,15,16,17]. The motivation of TDA comes from the fact that there are discrepancies in geometrical and topological information in an abstract space formed by the data point clouds. In 3-D object recognition tasks, the point clouds lie on the surface of the objects, and TDA-based Mapper framework contributes to the recognition tasks [18]. Later in diabetes research, TDA techniques were successfully used in identify a type-2 diabetes subgroup through patient similarity analysis [19]. Also, TDA contributes to bimolecular data analysis, namely multidimensional persistence for protein folding analysis [20]. In [21], the multivariate data analysis of cultural heritage was studied. Among these works, studying the data with TDA tools delivers novel insights to compare to the traditional tools.

Recently in signal analysis tasks, TDA showed great potential for pattern classification and outlier detection tasks [22,23,24,25,26], than the traditional statistical-based methods. Motivated by these works, the main contribution of this work includes:

1. We propose a TDA-inspired nonlinear analysis framework for analyzing the nonlinear gait dynamics as a vibrant tool for classification of people with and without neuro-degenerative diseases. This started with building topological feature extraction models in the form of persistence landscape.
2. As a pioneer study on using topological-based disease classification, a novel nonlinear dynamics analysis methodology developed and verified with a public gait disease datasets comprising of time series signals of patients with different neuro-degenerative diseases.

For readers convenience, we structure the rest of the paper as follows:

1. The proposed nonlinear analysis framework with related technique theory backgrounds’ introductions are illustrated in Section 2;
2. Details of TDA procedures such as embedding gait stride signals into time series into 2-D space point clouds, building topological features, and implementation of classifiers are discussed in Section 3.
3. Results of the proposed methodology when used with the decision tree, random forests, k-nearest neighbor and naive Bayesian classifiers are presented in Section 4.
4. Finally, discussion of the results, and conclusion of the study including some directions of future research are introduced in Section 5 and Section 6.

## 2. Methods

### 2.1. Framework of Topological Gait Nonlinear Analysis

**Figure 1:**
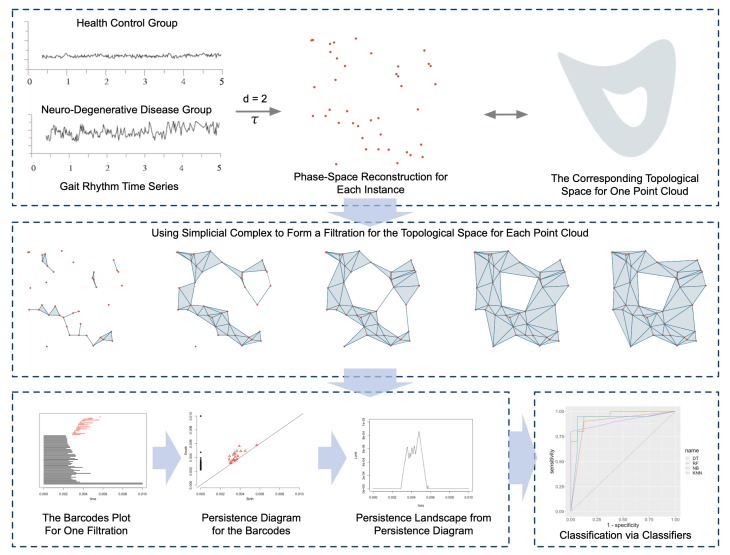
Topological Gait Analysis Framework: firstly, each time series is pre-processed and segmented into an equal-length time series; secondly, with time delay embedding the corresponding data point clouds are generated, each with one corresponding topological space; thirdly, the topological data analysis techniques are used to study the point clouds; finally, the topological features are extracted as a novel representation of the time series, thus used in the classification oriented machine learning framework for disease sensing.

The topological gait analysis for the gait series are composed with the following stages, which are briefly illustrated in Figure 1. The framework includes six stages as following:

1. Phase-Space Reconstruction: the time series are embedded into an abstract space, namely the time series sequences are transformed into one 2-D point cloud, with which resampling was adopted to reduce the scale. The point clouds are considered as in some abstract space;
2. Filtration Extraction: the point clouds are studied with the simplicial complex theory, the filtrations are achieved for each corresponding space from the point clouds;
3. Barcodes Generation: from the filtration the birth–death intervals for each homology are extracted, which can be illustrated by the Barcodes;
4. Persistence Diagram Generation: the Barcodes can be represented by the persistence diagrams, from which the persistence landscape features can be acquired;
5. Persistence Landscape Feature Acquiring: the persistence landscape features are used as the input as a Gaussian Naive Bayesian classifier toward the classification task.
6. Classification: the persistence landscape features are used as the input of classifiers toward the classification task.

### 2.2. Gait Data Point Cloud Construction

The time series are time-ordered observation sequences from the corresponding underlying dynamical systems. Time-series analysis problems can thus be transformed into the studies of dynamics behind the observed time-ordered data [27]. The phase-space reconstruction method is such a kind of tool developed for dynamics analysis towards time series analysis. Even though the reconstructed space by phase-space reconstruction is not identical to the real system, the abstract spaces reveal the system properties because of the topological equivalent [28]. Typical applications for biomedical signal analysis are [29] for multiple heartbeats ECG analysis, and [30] for streaming time series analysis with the application to motion analysis. In this work, we first convert the gait time series into gait data point cloud as the phase space reconstruction, using time-delay embedding.

Mathematically, we consider the time-series signal sequence $f\left(n\right),n\in Z^{+}$. For a time delay embedding operation, let $S\in Z^{+}$ as the parameter of delay step, the dimension of the topological space to be embedded into is $d\in Z^{+}$, then the time delay embedding (DE) at $t\in Z^{+}$ can be illustrated as:

(1) DE(f,t;s,d)={f(t),f(t+s),⋯,f(t+(d−1)s)}

The reconstruction of the phase space could convert the signals into higher dimensional abstract phase-space, which approximates the phase-state of the real dynamics. A central problem in doing the phase space reconstruction is the determination of the parameters of time delay parameter *τ* and the embedded dimension *d*. Appropriate *d* and *τ* could approximate the dynamics better which could help for further analysis. Estimating good values for *d* and *τ* is quite challenging, and lots of methods have been developed to choose the parameters [27,31]. In practice, *τ* was determined first and then *d*. However, in this work we set the dimension of the embedding space as *d* = 2. As stated in former study of [25,27], a larger *d* does not necessarily increase the classification performance. For the determination of *τ*, we adopt a heuristic by evaluating the results for classification performance. With time delay embedding, the time-series for each subject are converted into 2-D point clouds.

### 2.3. Topological Gait Analysis

The data point clouds are considered as lying on some topological spaces. The task of analyzing the features of the time series from different categories is converted into the study of the topological spaces properties, which the data point clouds are lying on. The TDA methods extract information from the topological properties of the data point cloud using the simplicial complex theory. With simplicial complexes, the topological summaries Barcode, and persistence diagram are extracted with persistence homology theory. Based on the topological summaries the persistence landscape features are used in statistical learning framework together with the random forests classifier, which was used as a descriptor to distinguish the neuro-degenerative diseases with health control subjects group.

#### 2.3.1. Simplicial Complex

**Figure 2:**
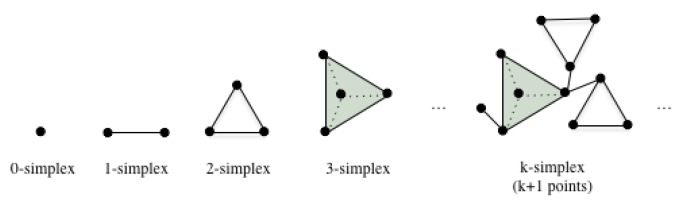
Simplex and simplicial complex.

A topological space can be understand as a set of points sampled from some $R^{n}$ with neighbors. The neighbor relations could be used to study the connectivities in a graph. In algebraic topology, the basic building blocks is simplicial complex, which is a data structure that explains how to ‘glue’ a topological space with simplices, i.e., points, edges, triangles, tetrahedra, and even their higher-dimensional generalizations. The definition of simplex is Definition 1 and the illustration can be found in Figure 2. Generally, a *k*-dimensional simplex is defined by *k* + 1 vertices.

**Definition 1** (Simplex). *Given a family of sets, any subset of cardinality k+1 is called a k-simplex. The vertices can be considered as 0-simplices, edges for 1-simplices, and triangular faces as 2-simplices, etc.*

A simplicial complex is a collection of simplices together with their faces. The face of a simplex is defined by the vertex subset. For instance, the faces of an edge of two vertices are the two endpoint vertices, and the edge itself. The faces of a 2-simplex (triangle with 3 vertices) includes the three vertices, the three edges, and the triangle itself. The collection with a simplex and its all faces is called a simplicial complex as defined in Definition 2.

**Definition 2** (Simplicial Complex). *A simplicial complex K is a finite collection of simplices such that*

1. *any face of σ∈K is also in K*
2. *for*$\sigma_{1},\sigma_{2}\in K$, $\sigma_{1}\cap \sigma_{2}$*is a face of both*$\sigma_{1}$*and*$\sigma_{2}$

#### 2.3.2. The Rips Complex and Graph Filtration

The task for extracting the topological signatures from the vertices set, i.e., the data point cloud. There are different ways to build the complex to approximate the topological space, such as Vietoris–Rips complex [32,33], Graph-induced complex [34], and Sparsified C$\overset{ˇ}{e}$ch complex [35]. In this work we adopt the Vietoris–Rips complex, which sometimes was called Rips complex for simplicity.

**Figure 3:**
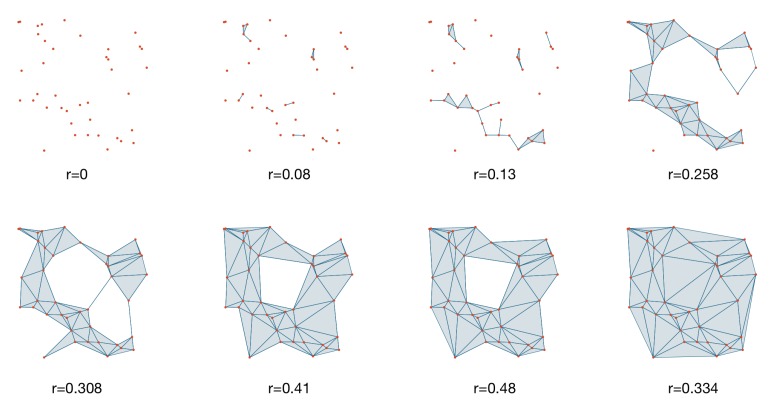
Persistent homology.

For the building process of Rips complex, here we use a similar illustration as in [36]. We consider the distance d(x,y) for vertices *x* and *y*. For the simplicial complex theory we consider a *ϵ*-ball with the radius ϵ=0 for the vertices set namely the original data point cloud. Intuitively, if we increase the radius *ϵ* gradually, when the distance between any two vertices d(x,y) is less than *ϵ* the edge appears. Then it is easy to think that for three vertices, the *ϵ*-balls intersect mutually (not totally merge together), a triangle appears; while for three vertices a tetrahedron emerges, and higher dimensional simplices whenever possible. We consider the process in Figure 3, the original point cloud is vertices without any edges with *ϵ* = 0. When *ϵ* = 0.08, any vertices with distance less than 0.08 are connected, thus some edges appear. Keeping on increasing *ϵ*, finally, any two points are linked, thus generating a fully connected graph (*r* = 0.334), and triangles are generated as well. At each stage, the homology of the simplicial complex is changing, some components born while some died. For instance in Figure 3, the quadrilateral hole is born during an event time before e = 0.41 and dies at the event time after e = 0.48. Then we can say the hole is alive between *ϵ* = 0.41 and *ϵ* = 0.48. Similarly, there are much more higher-dimensional holes when we consider a more complicated point cloud in practical applications.

Mathematically, consider data point cloud $X=\left\{x_{1},\ldots ,x_{n}\right\}\subset R^{n}$, the associate topological space using the Rips simplicial complex construction, which could be denoted by R(X,ϵ) in which R stands for Rips. The process in Figure 3 can be considered as the sequence:

(2) $$R\left(X,\epsilon_{0}\right),R\left(X,\epsilon_{1}\right),\cdots R\left(X,\epsilon_{n}\right)$$

and when the *ϵ* increase, the previous Rips complex is included in the subsequent one, i.e.,

(3) $$R\left(X,\epsilon_{0}\right)\subseteq R\left(X,\epsilon_{1}\right)\subseteq \cdots \subseteq R\left(X,\epsilon_{n}\right)$$

where $\epsilon_{0}\leq \epsilon_{1}\leq \cdots \leq \epsilon_{n}$. The increasing sequence of *ϵ* value produces a *filtration*: given a set *X*, the *K*-simplex $\left\{\sigma_{1},\sigma_{2},\ldots ,\sigma_{k+1}\right\}$, then we have the definition of filtration:

**Definition 3** (Filtration). *A filtration of a (finite) simplicial complex K is a sequence of sub-complexes such that*

1. $\emptyset =K^{0}\subset K^{1}\subset K^{2}\cdots \subset K^{m}=K$
2. $K^{i+1}=K^{i}\cup \sigma^{i+1}$ where $\sigma^{i+1}$ is a simplex of K

The algorithm of how to build the filtration in detail can be refereed to [37].

#### 2.3.3. Barcode and Persistent Diagram

**Figure 4:**
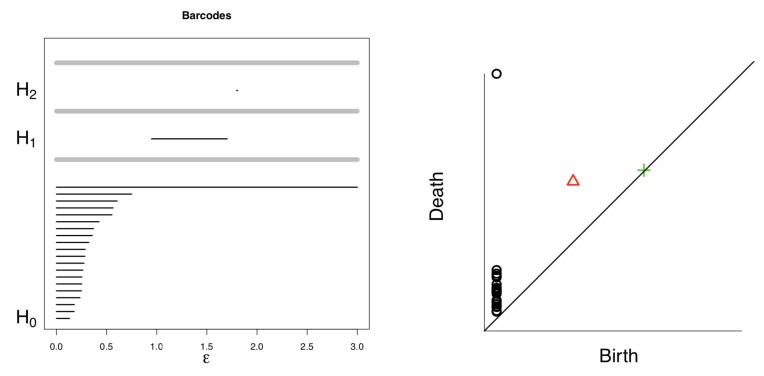
A barcode plot and the corresponding persistent diagram with *H*_0_, *H*_1_ and *H*_2_ [38].

So far the gait dynamics time series are converted into data point clouds, each with a series of Rips complex namely graph filtration. The filtration topological characteristics are changing while the *ϵ* changing, the process could be visualized using the barcodes. The topological feature is a bar plot which starts and ends when a topological property appears and disappears. For example in Figure 3, the sole hole in $K_{6}$ when *ϵ* = 0.41 appears and disappears at $K_{7}$ when *ϵ* = 0.48, the barcode for this hole is born at *ϵ* = 0.41 and dies at *ϵ* = 0.48. With all such “holes” and the corresponding **born time and death time, the topological features are represented. Then each topological feature of** the point cloud has a birth time $b_{\alpha }$ and death time $d_{\alpha }$. The features set with birth and death times is $\left\{\left(b_{1},d_{1}\right),\left(b_{2},d_{2}\right),\ldots ,\left(b_{m},d_{m}\right)\right\}$. This set can be illustrated in the Barcode plot and persistence diagram as in Figure 4.

#### 2.3.4. Persistence Landscape

Some applications directly consider the barcode as features [26], i.e., directly use the barcode intervals or statistical parameter of barcodes as input of the classifier in the machine learning task. Some other applications of barcode and persistence diagram are using Bottleneck and Wasserstein distance for comparison the topological similarity between persistence diagrams, for example in protein binding analysis [20]. Other essential related works are distance-based signal classification [22,39].

In this study, we further processed the barcode information to derive the persistence landscape as the features to be used in the classification task. We adopted Wasserstein distance for the gait dynamics application. Mathematically, the persistence diagram $P_{k}$ encoded from the *k*-dimensional homology *α* information in all scales. As the last section described, the homology *α* was “born” at $b_{\alpha }$ and “dies” at $d_{\alpha }$, which makes a pair $\left(b_{\alpha },d_{\alpha }\right)$ and pair set $\left\{\left(b_{1},d_{1}\right),\left(b_{2},d_{2}\right),\ldots \left(b_{m},d_{m}\right)\right\}$. We use $z_{\alpha }\in R$ to denote each pair and consider such pair as a point. Then the barcode graph can be transformed into a persistence diagram graph with birth indices on horizontal axis and death indices on the vertical axis as in Figure 4.

Persistence diagram are encoded from the information of the homology at all scales as barcode illustrations. The axes of the persistence diagram are birth indices (horizontal axis) and death indices (birth axis). The Wasserstein distance is often used as a standard metric to analysis the persistence diagram space as:

(4) $$W_{p}\left(P_{k}^{1},P_{k}^{2}\right)=inf_{\phi }[\sum_{q\in P_{k}^{1}}||x-\phi \left(x\right){\left|\right|}_{\infty }^{p}{]}^{\frac{1}{p}}$$

The equation is termed as the *p*-th Wasserstein distance, when p=∞ the metric is known as Bottleneck distance. Based on the Wasserstein metric, a representation termed Persistence Landscape has been proposed for statistical analysis by [40]. For each birth–death point $\left(b_{\alpha },d_{\alpha }\right)\in P_{k}$, a piecewise linear function:

(5) $$f\left(b_{\alpha },d_{\alpha }\right)=\left\{\begin{array}{ccc} x-b_{\alpha }, & \;if & x\in \left(b_{\alpha },\frac{b_{\alpha }+d_{\alpha }}{2}\right]\\ -x+d_{\alpha }, & \;if & x\in \left(\frac{b_{\alpha }+d_{\alpha }}{2},d_{\alpha }\right)\\ 0, & \;if & x\notin (b_{\alpha },d_{\alpha }) \end{array}\right.$$

with which a sequence of functions *λ* can be given by:

(6) $$\lambda_{k}\left(x\right)=k-max\left\{f_{\left(b_{\alpha },d_{\alpha }\right)}\left(x\right)|\left(b_{\alpha },d_{\alpha }\right)\in P_{k}\right\}$$

where the *k*-max denotes the *k*-th largest value of a function. Persistence landscape lies in a vector space, it is easy to combine with tools from statistics and machine learning, more theory description of persistence landscape can be referred from [40,41,42]. For intuition, as Figure 5 from [43] illustrated, the persistence landscape is one rotation from the persistence diagram.

#### 2.3.5. Pattern Recognition

We perform the recognition task with the persistence landscapes generated from the point clouds, In this study, we use a one-vs-one binary classification setting for the healthy control group with each of the amyotrophic lateral sclerosis group, Huntington’s disease group, and Parkinson’s disease group. In order to validate the discrimination ability for the proposed topological features, we adopt the following classifiers for the binary classification tasks:

1. Naive Bayes classifier (NB): Naive Bayes is one simple supervised machine learning classifier based on the Bayes theorem with an independence assumption between the feature. The Gaussian naive Bayesian classifier is used in this study.
2. Decision Tree (DT): Decision tree classifier searches for the nonlinear relationships between the inputs and outputs of the system. The DT classifier separates the features into branches and nodes.
3. Random Forests classifier (RF): One random forest classifier includes a set of decision trees which is widely used as one baseline classifier in the classification tasks.
4. K Nearest Neighbor (KNN): K nearest neighbors stores all available cases and classifies new cases based on a similarity measure, which is a simple unsupervised classifier. Here we set the neighbor number as 3.

For each binary classification task, we consider the accuracy, sensitivity, specificity, and AUC score as the performance assessment parameters (details as in Section 4.3).

## 3. Experiments

### 3.1. Materials

In this study, we adopt the Gait Dynamics in Neuro-Degenerative Disease Dataset [11] from the Physionet [44] database. The dataset was proposed for a better understanding of pathophysiology in neuro-degenerative diseases. It includes 16 healthy control subjects, 15 patients with Parkinson’s disease, 20 Huntington’s disease, and 13 subjects with amyotrophic lateral sclerosis. We use HC, ALS, HD, and PD as the abbreviations. A detail description of the subjects’ clinical information with age, gender, height, weight, walking speed, disease severity of PD and HD, and duration of ALS was included in the dataset, the illustrations of HC, PD, HD, and ALS group are given in Table 1, Table 2, Table 3 and Table 4 respectively.

**Figure 6:**
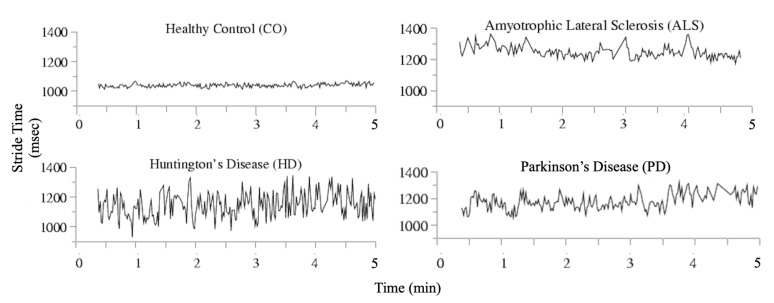
An illustration for the gait dynamics time series [11].

The raw data of the database were obtained using force-sensitive resistors, with the output roughly proportional to the force under the foot. The stride-to-stride measurements of footfall contact times has been derived from the signals, which includes left stride interval, right stride interval, left swing interval, right swing interval, left stance interval, right stance interval, and double support interval. As well the left/right swing interval percentages of stride, left/right stance interval percentages of stride, and double support interval percentages of stride are extracted. The detailed description and experiment settings of the dataset can be referred to [11,45]. Illustrations for each group in the dataset are shown in Figure 6.

### 3.2. Outlier Processing

The startup effect of the gait acquisition was minimized with removal of the first 20 s in the dataset as in the previous work of Hausdorff et al. [2]. However, according to the experiments, the subjects are asked to perform a 5-min walking which includes several turn-back and body rotations. This process could lead to outliers in the stride intervals, here we realized the outlier detection and replacement strategy as proposed in [2]. For each time series, a median value and the standard deviation were calculated as $\hat{\mu }$ and *s*, respectively. Assume the length of the time series is *N*, then for each value $t_{i},i=1\ldots N$ in the time series, the outlier process was computed as in Equation 7:

(7) $$t_{i}=\left\{\begin{array}{cc} \hat{\mu } & \mathrm{if}\;t_{i}>\hat{\mu }+2s\\ \hat{\mu } & \mathrm{if}\;t_{i}<\hat{\mu }-2s\\ t_{i} & \mathrm{otherwise}. \end{array}\right.$$

We can see that all outliers are replaced with the median value of the stride interval time series (large outliers could make the mean of the time series quite large).

### 3.3. Experiments Setup

After the replacement of time intervals, we use the right-foot stride-interval signals from the HC, ALS, HD, and PD groups in the experiments. In this work, we consider the classification performances in a one-vs-one setting as in former studies like [10,46]. The involve classification groups are the HC, ALS, HD and PD groups, we consider the binary classification performance of HC group versus ALS group, HC group versus HD group, and HC group versus PD group.

We use the methodology proposed in Section 2 to build the topological feature sets for each subjects. The right-foot-stride time series for each sample are transformed into persistence landscapes. Then three binary classification tasks are performed using a leave-one-out cross-validation (LOO-CV) strategy [47] to evaluate the models using the classifiers. Then we have 16 persistence landscape feature vectors for HC group, 13 for ALS, 20 for HD, and 15 for PD. For each classification task, we have data samples from the HC group and its corresponding abnormal group. First, we leave one sample out from the classification data set, and use the remaining samples to train the classifier. Second, the trained classifier was used to predict the label of the sample that was left out. Third, the process was performed with different leave-out choices of samples in the data set.

### 3.4. Performance Assessment

For the binary classification tasks, the confusion matrices are calculated as illustrated in Table 5, where TP stands for true positive; TN stands for true negative; FN stands for false negative; FP stands for false positive. A confusion matrix contains the information about actual labels and predicted labels by the classification system.

From the confusion matrix, we can get the accuracy, sensitivity and specificity parameters as follows respectively:

(8) $$accuracy=\frac{TP+TN}{TP+FP+TN+FN}$$

(9) $$sensitivity=\frac{TP}{TP+FN}$$

(10) $$specificity=\frac{TN}{FP+TN}$$

Moreover, for comparison with other related works we also consider the receiver operating characteristic curve (AUC), which was generated from the confusion matrix information, where an AUC of 1 means a perfect test and an AUC of 0.5 represents a random guess. A higher AUC score means the model is better than the lower ones.

## 4. Results

### 4.1. Time Delay Embedding

The embedding process converts the time series into data point clouds in abstract phase space. An embedding dimension = 2 is used in this study, this strategy can drastically reduce computational cost. Moreover, recent literature states that a higher dimension would not significantly increase the performance for the final classification tasks [25]. The choice for the time series phase-space reconstruction is different from the studies of searching for the best embedding parameters in [27]. The best reconstruction dimensions and time delay parameters can be achieved with AMI an AAI technique, as illustrated in previous studies [31]. However, here we set a determined embedding dimension as *d* = 2, and we consider a heuristic method to test several candidates *τ* for the classification. In Figure 14 we used different *τ* values in the time delay embedding, it is observed that the reconstructed space loses information to distinguish pattern differences when using too large, or too small *τ* values. Then we only use embedding time delay parameters of τ=5,6,7,8 as typical choices of the holistic framework for neuro-degenerative disease classification in the further discussion. The corresponding results are illustrated in Figure 11, Figure 12 and Figure 13, and further discussed in Section 5.

**Figure 7:**
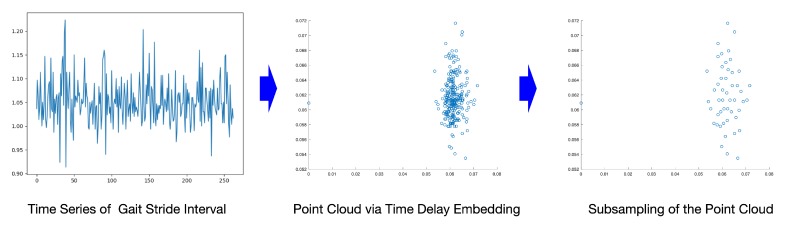
An illustration for time delay embedding subject HD #6 (d=2,τ=5). Left: original stride interval time series (263 interval numbers). Middle: point cloud via time delay embedding (259-point cloud in the 2-D space). Right: subsampling of point cloud (50-point cloud).

After the reconstruction process, we convert the gait time series into data point clouds. In the experiment, each subject walk for 5 min at different speeds. The speed differences could lead the sampling data points to have a different number of walking stride intervals. After performing time delay embedding, the corresponding data point clouds’ scales are different. In order to make the data sample size consistent, we use a resampling technique based on [48]. This technique approximates the original point cloud, which keeps the shape and topological information on a smaller scale. Here we resample the data point clouds into 50 points, which was used for the following topological feature extraction. In Figure 7, we have the illustration for the embedding process and subsampling for one subject from the HC group. Here we resample the points cloud into a 50-point scale, i.e., transform each time series into a 50-point large 2-D data point cloud.

### 4.2. Topological Features

After the phase space reconstruction, the stride-interval time series were transformed into data point clouds in 2-D space. With the points, we extract the topological features with the techniques described in Section 2. The topological features extracted from each group are illustrated for intuition in the following sections.

#### 4.2.1. Barcodes

**Figure 8:**
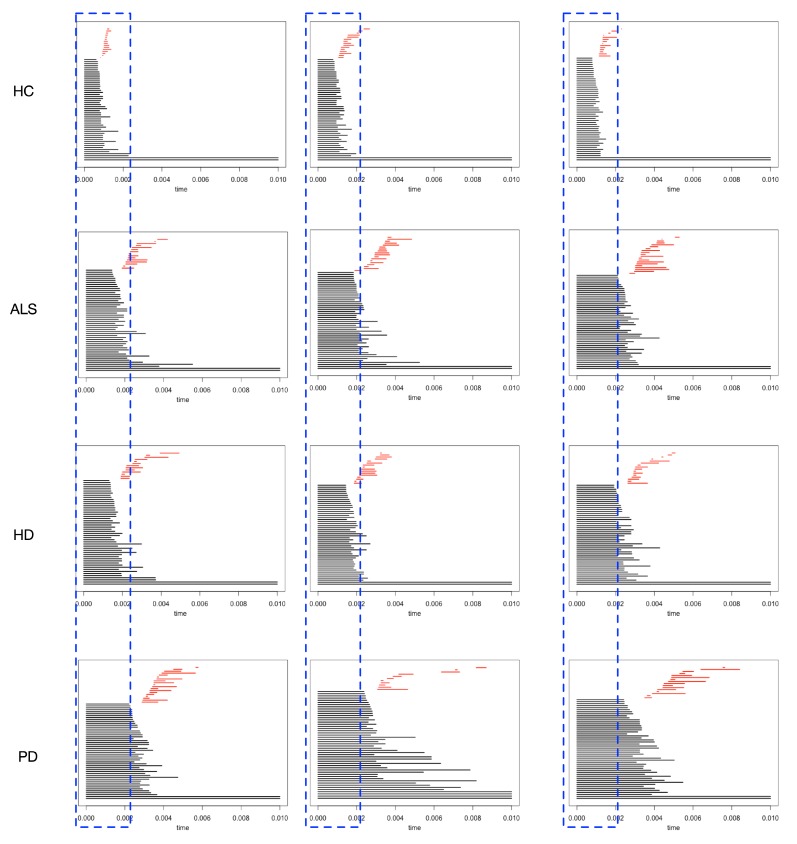
An illustration for the barcodes for each group. First row for the HC group; second row for ALS group; third row for the HD group; fourth row for the PD group. With the blue box-based indicator, we can see that the red bars’ positions are different in the HC group (first row) to the rest, i.e., the 1-dimensional homologies’ durations (appear earlier and vanish earlier for the red bars) in the HC group are different from abnormal classes.

With the technique of persistence homology, gradually increase the radius of the points in the data point clouds, homologies are generated and vanished as the radius increases. In Figure 8, the 0-dimensional homologies (the dark bars), and 1-dimensional homologies (the red bars) are illustrated. Each row of Figure 8 illustrates one class (normal and one of abnormal types).

Our main task is to distinguish the neuro-degenerative disease stride interval type from the healthy control subjects. Thus, we mainly consider the discrepancies between the abnormal class and the healthy control class, i.e., the difference between the first row and the rest. The placed rectangular boxes with equal width show that the red bars in the first row are earlier than the rest rows. The red bars are the lifetime for the 1-dimensional homologies, and then the barcodes can be used as features to distinguish the abnormal groups from the healthy control group. In [26], using the length of the bars in the Barcodes from the point cloud as the time-of-life features was proposed in classification tasks. Likewise, in [23], one typical bar in the Barcodes (lifetime of one typical homology) is one marker for the variation for detection tasks.

#### 4.2.2. Persistence Diagrams

**Figure 9:**
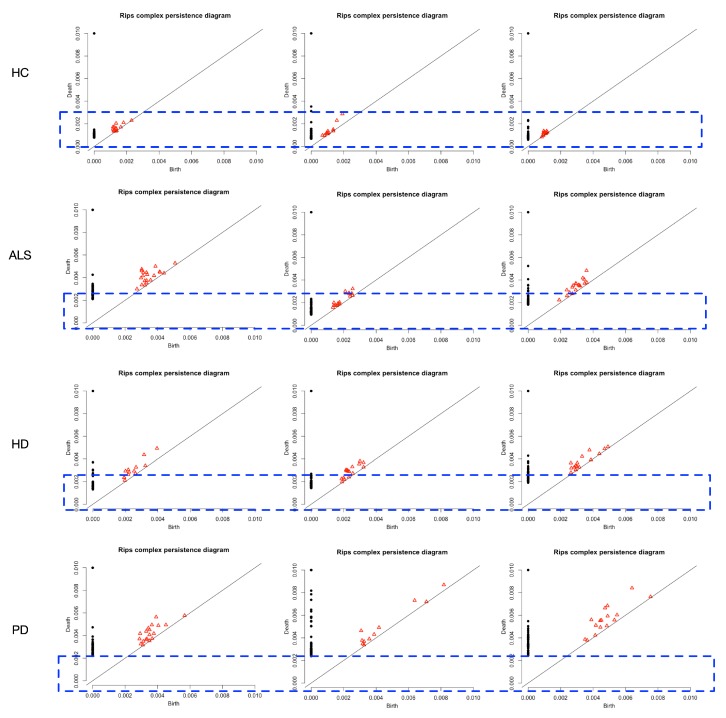
An illustration for the persistence diagrams for each group. First row for HC; second row for ALS; third row for HD; fourth row for PD. Each sub-figure corresponds to the sub-figure in Figure 8. With the blue box-based indicator, we can see that in the HC group, the red triangles are inside of the box, while the others are not.

Persistence diagrams plot is an alternative graphical way to represent barcodes. It shares the same information as the barcodes. For each barcode plot of the point clouds from the gait interval time series, a corresponding persistence diagram can be accomplished, i.e., the gait dynamics for each individual is illustrated by one persistence diagram. As illustrated in Figure 9, the points illustrate the 0-dimensional homologies while the red triangles represent the 1-dimensional homologies. The horizontal axes in Figure 9 represent the birth time and vertical ones for death time. From the placed rectangular boxes with equal heights, we can see that in the first row the red triangles are within the boxes, while the rest are out of the corresponding box.

#### 4.2.3. Persistence Landscape

**Figure 10:**
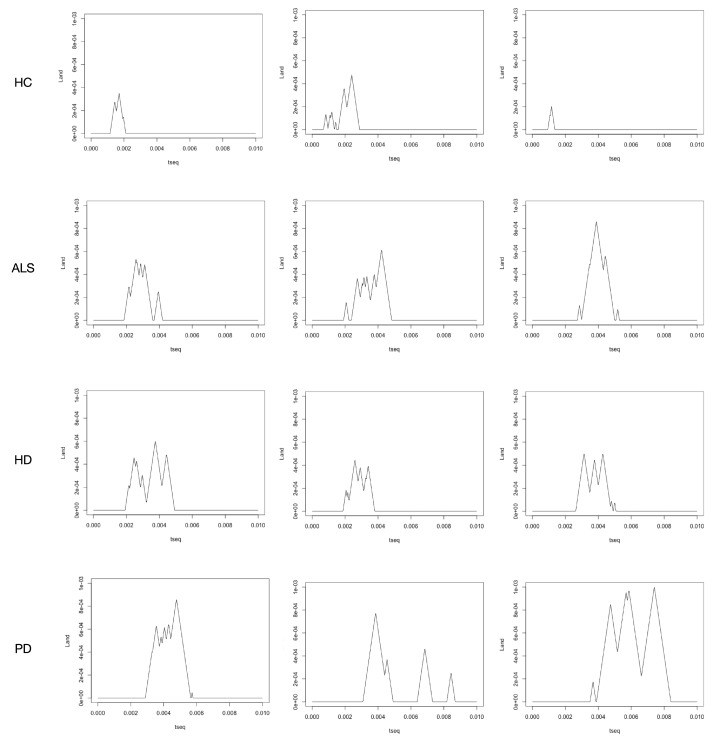
The persistence landscapes corresponded to Figure 8 and Figure 9.

The persistence landscapes for each persistence diagram are constructed. This transformation formally has no information loss, while the persistence diagrams are mapped into a functional space, which makes the machine learning and statistical tools available. Here we consider the transformed persistence landscape as one feature set for the disease classification task. We build the classification system using the persistence landscape features illustrated in Figure 10 for disease classification.

From the barcodes plot and persistent diagram, we can see that there is a significant difference between the 1-dimensional homology-based features. Thus, here we only consider the 1-dimensional homology persistence landscape. Figure 10 illustrates the corresponding 1-dimensional homology persistence landscapes of Figure 8 and Figure 9.

### 4.3. Pattern Recognition

For the different classification tasks, the optimal time lag parameters are different. Here we keep the parameters of the classifiers fixed and set the time delay parameter τ=5,6,7,8. For the decision tree classifier, the maximal depth for the tree is set as 5. For the random forest classifier, the tree number is set as 1200. For the k-nearest-neighbor classifier, the number of neighbors is set as 3. The classification tasks are performed with the Python package scikit-learn [49], the none-mentioned parameters are used with default settings. The results are illustrated in the following sections.

**Figure 11:**
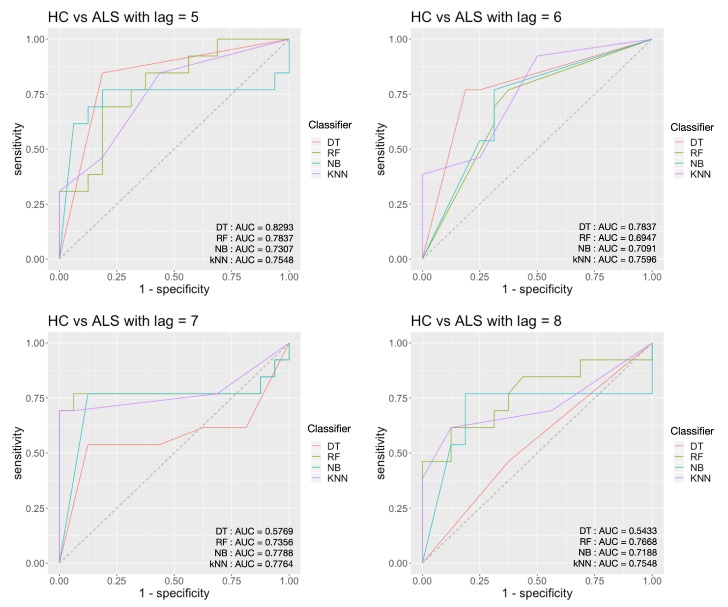
Classification results for HC vs. ALS with an embedding time delay lag of 5, 6, 7 and 8.

For the HC vs. ALS task, from Figure 11, we can see that the highest AUC score is 0.8293 and achieved by the decision tree classifier with persistence landscape when *τ* = 5. The corresponding accuracy is 82.76%, and sensitivity is 81.25%, and the specificity is 84.62%.

**Figure 12:**
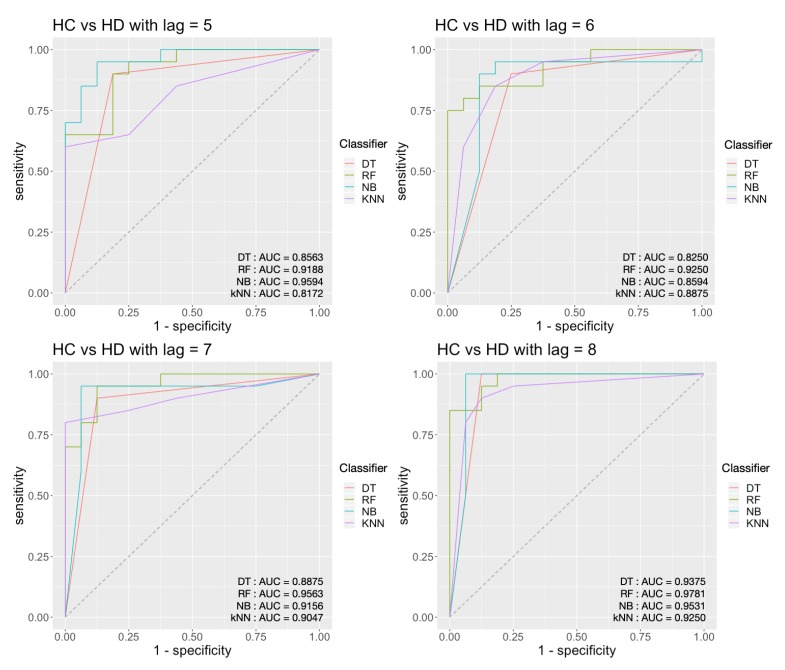
Classification results for HC vs. HD with an embedding time delay lag of 5, 6, 7 and 8.

For the HC vs. HD task, from Figure 12, we can see that the highest AUC score is 0.9781 and achieved by the decision tree classifier with persistence landscape when *τ* = 8. The corresponding accuracy is 91.67%, and sensitivity is 87.5%, and the specificity is 95.00%.

**Figure 13:**
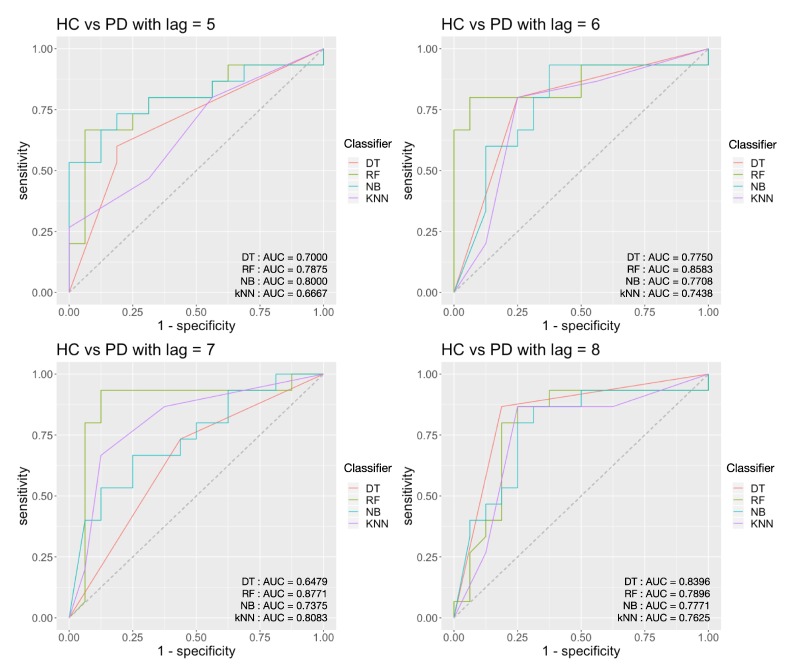
Classification results for HC vs. PD with an embedding time delay lag of 5, 6, 7 and 8.

For the HC vs. PD task, from Figure 13, we can see that the highest AUC score is 0.877 and achieved by the decision tree classifier with persistence landscape when *τ* = 7. The corresponding accuracy for the AUC score is 90.32%, and sensitivity is 87.5%, and the specificity is 93.33%.

## 5. Discussion

### 5.1. Nonlinear Analysis of Gait Dynamics

The phase space is an abstract multidimensional space, which is used to graphically represent all the possible states of a dynamical system [50]. The reconstructed phase spaces are topologically equivalent to the original system and, hence, can recover the nonlinear dynamics of the system. Previous studies proposed different parameters for the nonlinear dynamics analysis. Lyapunov exponent was proposed as one quantitative measure of the divergence or convergence average rate of the trajectory composed of points in phase state space [51]. In the concept of fractal dimension analysis, the data points were considered as geometrical objects, which possess a definite dimension. For example, a point, a line, and a surface have dimensions of 0, 1, and 2, respectively [52]. The recurrence plot analysis, and Poincaré plot analysis [53] enables the visualization of the evolution of a dynamical system in the phase space, which are useful for the identification of the hidden patterns. These methods have been adopted in the gait nonlinear dynamics analysis in [46].

The information contained in the point clouds in the phase state space can be re-express in the topology way. In this work, we consider the data point clouds as topological objects, from which the patterns are discovered. From the nonlinear dynamics angle, the proposed framework provided a novel method for the phase space analysis, namely the TDA method. At the same time, the patterns from the gait dynamics are illustrated with the persistence landscape developed from the topological method, from which we perform the neuro-degenerative disease analysis. In summary, the proposed work brought a two-fold development: a novel nonlinear dynamics analysis framework, and a gait dynamics classification system toward disease classification.

### 5.2. Time Delay Embedding

**Figure 14:**
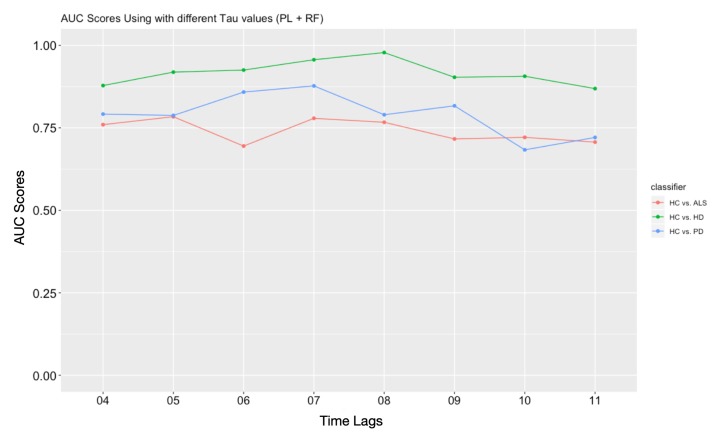
The AUC scores of the wholistic system using different time lag parameters in generating topological featurs.

The topological features are based on the data point cloud generated by the reconstruction of the state space. Better reconstruction of the phase state space from the time series could better reflect the real pattern of the dynamical system. There are lots of principles has been proposed to solve this problem, such as the average mutual information, to determine an appropriate dimension and time lag parameter. However, since our main purpose is to get better performances for the wholistic system, an optimal parameter for a specific time series is different from the classification task here. In our work, we only consider a 2-D situation, so the time lag parameter for embedding has a great impact on TDA feature generation. So we adopt a heuristic strategy to search for the range of time lag within the range from 4 to 11 since the reconstruction lag should be neither too large nor too small. This process is illustrated in Figure 14, and we consider the wholistic classification performances with an AUC score.

### 5.3. Related Work and Comparisons

The present study demonstrated that the persistence landscape-based topological method is promising in gait variability analysis. Unlike the traditional methods, the presented framework considers the gait variability as different state behaviors under a fixed process. The right-foot-stride time series are converted into point clouds in the abstract space, as a characterization of the underlying system. We construct a persistence landscape-based feature set, as the systems’ representation for each subject. Sequentially, we used four classifiers to validate the distinguish ability of the extracted features. In Table 6, Table 7 and Table 8 we compared the results with some of the former works.

#### 5.3.1. HC vs. ALS

The empirical mode decomposition (EMD) method was proposed in [8], five types of time series of gait rhythm fluctuations were involved: stride time, swing time, stance time, percentage swing time, and percentage stance time. The best AUC score achieved with the EMD technique was 0.934 with the multi-layer Perceptron classifier in the HC vs. ALS classification. The phase synchronization and conditional entropy (PSCE)-based method was proposed in [54], with the same data source as in EMD methods in [8], the best AUC score was 0.824 with a multi-layer Perceptron classifier in the HC vs. ALS classification. Determine learning strategy using left and right swing intervals, and the left and right stance intervals was proposed in [55], with an accuracy of 89.66% in the HC vs. ALS classification.

Similarly to the the previous works, we evaluated our proposed topological-based features in the existing classification schemes and obtained relatively high accuracy. Specifically, employing the persistence landscape features for the classification task with decision tree yielded a comparable result of AUC = 0.829 with some existing methods. Although for the HC vs. ALS classification, the performance of the framework is a bit lower than those of the existing studies described above. However, this could be attributed to using only the right-foot-stride intervals as the data source in our study.

#### 5.3.2. HC vs. HD

The corresponding work using EMD in HC vs. HD task in [8] gives the best AUC score 0.900. Determine learning achieved an accuracy of 83.33%. With the PSCE parameter and a multi-layer Perceptron classifier, an AUC score of 0.959 was achieved. It is important to state that the proposed TDA-based framework has the best outperformance compared with when all the traditional statistical-based methods were used for the HC and HD classification. In fact, we achieved an AUC score of 0.978, which is higher than using the traditional approaches as used in the previous works. This shows that using the persistence landscape feature with a random forests classifier using can ensure a better classification of healthy and non-healthy subjects based on the right-foot-stride time series signals.

#### 5.3.3. HC vs. PD

In [7] a nonparametric Parzen-Window-based method was adopted to estimate the probability density functions of stride interval, swing interval, and stance interval time series. From this the gait rhythm standard deviation and a signal turns count (STC) parameter were derived as dominant features, with an AUC score of 0.952. The corresponding work using EMD in HC vs. PD task in [8] gives the best AUC score 0.949. With determine learning an accuracy of 87.10% was aquired. With the PSCE parameter and a multi-layer Perceptron classifier, an AUC score equal to 0.928 was achieved. In [56] with the same time series as this study, namely right-foot-stride intervals, a hidden Markov model was proposed for the classification task with an accuracy of 90.32% was achieved.

## 6. Conclusions

### 6.1. Conclusions

A TDA-inspired nonlinear dynamics analysis framework has been presented and applied to gait dynamics analysis from the healthy control subjects and three neuro-degenerative diseases patients. Firstly, the gait-based physiological time series are embedded into phase state space as data point clouds using time-delay embedding; then, the persistence landscape-based topological representations are extracted via TDA techniques; finally, the topological gait feature-based classification are performed to validate the distinguish ability. As a result, the TDA-based nonlinear dynamics framework shows good classification ability of physiological signals. The extraction of persistence landscape features from the data point cloud using TDA techniques fits well into the mechanism of the physiological dynamical system for capturing information from the time series data. Combined with the random forest-based classifier, the persistence landscape representation extracted from the data point cloud of right-foot stride intervals time series is promising for differentiating healthy control subjects from neuro-degenerative disease patients.

### 6.2. Limitations and Future Work

The presented results show that the topological representations perform well in the classification tasks. However, there are several limitations and unsolved problems when using this technique. Firstly, the optimization parameters in the phase space reconstruction for TDA feature generation are a challenge. In the previous work, the proposed parameters searching strategy for dimension and time-delay lag [31] were not designed in a recognition-oriented task. Secondly, the experimental subject numbers are limited. We need more tests for performing on a larger real-world dataset. Thirdly, we only consider the right foot stride interval time series. The topological gait nonlinear analysis with stride information, swing and stance information are promising for a full understanding of the gait dynamics.

## Figures and Tables

**Figure 5:**
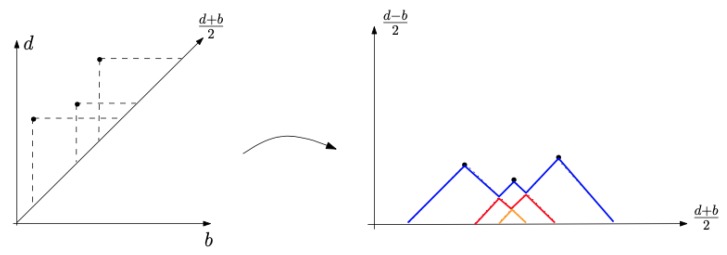
An example of transformation from a persistence diagram to the persistence landscapes [43]. Left: the horizontal axis in the persistence diagram is the birth time, while death time is on the vertical axis. Right: the horizontal axis is the average of the homologies’ birth death event times, and vertical axis for (d−b)/2.

**Table 1:** The subjects’ clinical information and interval number of the healthy control (HC) group.

ID	Age (Years)	Gender	Gait Speed(m/sec)	Severity Index	Stride Interval Number
HC # 1	57	F	1.33	0	259
HC # 2	22	M	1.47	0	241
HC # 3	23	F	1.44	0	255
HC # 4	52	F	1.54	0	267
HC # 5	47	F	1.54	0	250
HC # 6	30	F	1.26	0	270
HC # 7	22	F	1.54	0	260
HC # 8	22	F	1.33	0	261
HC # 9	32	F	1.47	0	275
HC # 10	38	F	1.4	0	277
HC # 11	69	F	0.91	0	269
HC # 12	74	M	1.26	0	244
HC # 13	61	F	1.33	0	251
HC # 14	20	F	1.33	0	249
HC # 15	20	F	1.19	0	198
HC # 16	40	F	1.33	0	250

**Table 2:** The subjects’ clinical information of the amyotrophic lateral sclerosis (ALS) group.

ID	Age	Gender	Gait Speed(m/sec)	Duration (Months)	Stride Interval Number
ALS # 1	68	M	1.302	1	194
ALS # 2	63	M	1.219	14	242
ALS # 3	70	F	0.853	13	215
ALS # 4	70	F	-	54	135
ALS # 5	36	M	-	5.5	205
ALS # 6	43	M	0.77	17	176
ALS # 7	65	M	1.302	9	159
ALS # 8	51	M	1.085	3	232
ALS # 9	50	M	0.899	54	212
ALS # 10	40	F	1.219	14.5	246
ALS # 11	39	M	1.283	7	229
ALS # 12	62	M	0.831	12	122
ALS # 13	66	M	0.832	34	183

**Table 3:** The subjects’ clinical information of the Huntington’s disease (HD) group.

ID	Age	Gender	Gait Speed(m/sec)	Severity Index	Stride Interval Number
HD # 1	42	M	1.68	8	310
HD # 2	41	F	1.05	11	225
HD # 3	66	F	1.05	4	232
HD # 4	47	F	1.4	2	268
HD # 5	36	M	1.82	10	263
HD # 6	41	F	1.54	8	263
HD # 7	71	M	1.05	2	232
HD # 8	53	F	1.26	9	256
HD # 9	54	F	1.26	12	270
HD # 10	47	F	1.05	4	220
HD # 11	33	M	1.26	11	239
HD # 12	47	M	1.19	8	258
HD # 13	40	F	0.56	5	167
HD # 14	36	F	1.4	12	255
HD # 15	34	F	0.56	3	217
HD # 16	70	M	0.56	5	190
HD # 17	29	F	1.19	12	248
HD # 18	54	F	0.98	2	252
HD # 19	59	F	0.98	1	243
HD # 20	33	F	-	9	238

**Table 4:** The subjects’ clinical information of the Parkinson’s disease (PD) group.

ID	Age	Gender	Gait Speed(m/sec)	Severity Index	Stride Interval Number
PD # 1	42	M	1.68	4	245
PD # 2	41	F	1.05	1.5	277
PD # 3	66	M	1.05	2	230
PD # 4	47	F	1.4	3.5	222
PD # 5	36	M	1.82	2	263
PD # 6	41	M	1.54	2	269
PD # 7	71	F	1.05	4	226
PD # 8	53	M	1.26	4	203
PD # 9	54	M	1.26	1.5	222
PD # 10	47	M	1.05	3	288
PD # 11	33	M	1.26	3	230
PD # 12	47	F	1.19	3	247
PD # 13	40	F	0.56	3	251
PD # 14	36	M	1.4	3	278
PD # 15	34	M	0.56	2.5	237

**Table 5:** The confusion matrix illustration.

Predicted Labels		
Actual	Positive	Negative
Positive	TP	FN
Negative	FP	TN

**Table 6:** Comparisons of AUC and leave-one-out cross-validation (LOO-CV) Results for classification of HC and ALS groups.

Methods	Accuracy (%)	Sensitivity (%)	Specificity (%)	AUC-Score
EMD + RF [8]	x	x	x	0.900
EMD + SLR [8]	x	x	x	0.859
EMD + MLP [8]	x	x	x	0.934
EMD + NB [8]	x	x	x	0.891
EMD + SVM [8]	x	x	x	0.906
DL [55]	89.66	92.31	87.50	x
PSCE + MP [54]	x	81.3	68.8	0.824
PSCE + RF [54]	x	93.75	75.0	0.789
PSCE + NB [54]	x	87.5	62.5	0.750
TDA: PL + DT	82.76	81.25	84.62	0.829
TDA: PL + RF	75.86	81.25	69.23	0.784
TDA: PL + NB	82.76	87.50	76.92	0.736
TDA: PL + KNN	86.21	100	69.23	0.776

**Table 7:** Comparisons of AUC and LOO-CV Results for classification of HC and HD groups.

Methods	Accuracy (%)	Sensitivity (%)	Specificity (%)	AUC-Score
EMD + RF [8]	x	x	x	0.885
EMD + SLR [8]	x	x	x	0.843
EMD + MLP [8]	x	x	x	0.878
EMD + NB [8]	x	x	x	0.898
EMD + SVM [8]	x	x	x	0.900
DL [55]	83.33	85.00	81.25	x
PSCE + MP [54]	x	100	85.00	0.910
PSCE + RF [54]	x	95.0	90.0	0.959
PSCE + NB [54]	x	95.0	80.0	0.920
TDA: PL + DT	94.44	87.50	100	0.938
TDA: PL + RF	91.67	87.5	95.00	0.978
TDA: PL + NB	83.33	100	70.00	0.959
TDA: PL + KNN	88.89	87.50	90.00	0.925

**Table 8:** Comparisons of AUC and LOO-CV Results for classification of HC and PD groups.

Methods	Accuracy (%)	Sensitivity (%)	Specificity (%)	AUC-Score
*σ* & STC + LS-SVM [7]	90.32	x	x	0.952
HMM [56]	90.32	93.33	87.50	x
EMD + RF [8]	x	x	x	0.865
EMD + SLR [8]	x	x	x	0.949
EMD + MLP [8]	x	x	x	0.910
EMD + NB [8]	x	x	x	0.875
EMD + SVM [8]	x	x	x	0.906
DL [55]	87.10	86.67	86.50	x
PSCE + MP [54]	x	100	81.3	0.928
PSCE + RF [54]	x	93.8	87.5	0.910
PSCE + NB [54]	x	87.5	81.3	0.898
TDA: PL + DT	83.87	81.25	86.67	0.840
TDA: PL + RF	90.32	87.50	93.33	0.877
TDA: PL + NB	77.42	100	53.33	0.800
TDA: PL + kNN	77.42	87.50	66.67	0.808

## Abbreviations

The following abbreviations are used in this manuscript:

TDA	Topological Data Analysis
HC	Healthy control
PD	Parkinson’s diseases
ALS	Amyotrophic lateral sclerosis
HD	Huntington’s disease
